# Adsorption Mechanism of Novel Porous Materials in Wastewater Treatment: A New Open Special Issue in Materials Science

**DOI:** 10.3390/ijms232314879

**Published:** 2022-11-28

**Authors:** Po-Hsiang Chang

**Affiliations:** 1 College of Resources and Environment, Fujian Agriculture and Forestry University, Fuzhou 350002, China; tectonicion@yahoo.com.tw; 2 Department of Soil and Environmental Sciences, National Chung Hsing University, 145 Xingda Rd., Taichung 40227, Taiwan

The special titled Adsorption Mechanism of Novel Porous Materials in Wastewater Treatment: A New Open Special Issue in *Materials* aims to publish high-quality research and review articles on the basic and applied science of porous materials and make great contributions to the understanding of metal-organic frameworks (MOFs) in removing pollutants from aqueous and soil environments.

Metal-organic frameworks (MOFs) are organic–inorganic composite functional materials usually referring to porous materials formed by metal ions or metal clusters and nitrogen- and oxygen-containing rigid organic ligands via the self-assembly process. MOFs are new materials that have developed rapidly in the field of coordination chemistry in recent years [1,2,3]. MOF materials have shown great application prospects in many fields due to its designable structure and easy chemical modification. MOF materials are widely used in the fields of gas storage and separation, fuel storage, catalysis, sensing, drug delivery, etc., possessing a broad application potential. For the practical application of MOF materials, the first consideration is that the integrity of their framework structure must be guaranteed to maintain the intended functions and properties. The stability of MOFs is affected by many factors, including the operating environment, metal ions, organic ligands, the geometry of metal–ligands, and the hydrophobicity of the pore surface [4,5]. Due to the weak coordination bonds, early synthesized MOF materials are prone toward skeleton collapse in aqueous environments, so they cannot be practically applied. In recent years, the problem of the poor stability of MOF materials has been gradually overcome. In previous reports, materials with good stability and that have been widely used mainly include some MOF, ZIF, MIL, UiO, and HKUST. Based on the structure and performance characteristics of MOF materials, in recent years, the field of environmental pollution control has used MOF and composite MOF materials for the removal of various pollutants, and these materials have shown broad application prospects [6,7]. In known studies, MOF materials have been used to remove heavy metals [8], radioactive pollutants [9], aromatic hydrocarbon pollutants [10], dyes [11,12], and gaseous pollutants [5,13].

In addition to heavy metals, organic pollutants are another important type of environmental pollutants. For biodegradable organic matter, biological methods are the most economical method to remove them. However, biological methods are incompetent for some macromolecular, refractory, and highly toxic organic pollutants. Because of its high efficiency and wide application, adsorption and catalytic degradation technologies are suitable alternatives to biological methods or can be used as a precursor to biological methods. Therefore, it has a wide range of applications in the treatment of organic pollutants, especially refractory organic pollutants. A large number of studies have shown that MOF materials can achieve the efficient removal of organic pollutants in water via adsorption or catalysis.

The research interest of this section on MOFs applications in pollutant removal includes but is not limited to the following: natural clay minerals such as zeolite, molecular sieve, metal composite material (MCM), and their organic and inorganic modifications; and other nanomaterials such as porous coordination polymers (PCPs), metal-organic porous materials (MOPMs), porous coordination networks (PCNs), or metal organic materials (MOMs) for the removal of a wide range of water pollutants (both inorganic and organic), including the contaminants of emerging concern from the environment.

## Data Availability

The data that support the findings of this study are openly available in references [1,2,3,4,5,6,7,8,9,10,11,12,13].
